# Altered dynamic functional connectivity of insular subdivisions among male cigarette smokers

**DOI:** 10.3389/fpsyt.2024.1353103

**Published:** 2024-05-16

**Authors:** An Xie, Yunkai Sun, Haobo Chen, Ling Li, Peng Liu, Yanhui Liao, Yonggang Li

**Affiliations:** ^1^ Department of Radiology, The First Affiliated Hospital of Soochow University, Suzhou, Jiangsu, China; ^2^ Department of Radiology, The People’s Hospital of Hunan Province (The First Affiliated Hospital of Hunan Normal University), Changsha, Hunan, China; ^3^ Center for Mind & Brain Sciences, Hunan Normal University, Changsha, Hunan, China; ^4^ Department of Psychiatry, Sir Run Shaw Hospital, School of Medicine, Zhejiang University, Hangzhou, Zhejiang, China

**Keywords:** insula, nicotine, subdivisions, fMRI, dynamic functional connectivity (dFC)

## Abstract

**Background:**

Insular subdivisions show distinct patterns of resting state functional connectivity with specific brain regions, each with different functional significance in chronic cigarette smokers. This study aimed to explore the altered dynamic functional connectivity (dFC) of distinct insular subdivisions in smokers.

**Methods:**

Resting-state BOLD data of 31 smokers with nicotine dependence and 27 age-matched non-smokers were collected. Three bilateral insular regions of interest (dorsal, ventral, and posterior) were set as seeds for analyses. Sliding windows method was used to acquire the dFC metrics of different insular seeds. Support vector machine based on abnormal insular dFC was applied to classify smokers from non-smokers.

**Results:**

We found that smokers showed lower dFC variance between the left ventral anterior insula and both the right superior parietal cortex and the left inferior parietal cortex, as well as greater dFC variance the right ventral anterior insula with the right middle cingulum cortex relative to non-smokers. Moreover, compared to non-smokers, it is found that smokers demonstrated altered dFC variance of the right dorsal insula and the right middle temporal gyrus. Correlation analysis showed the higher dFC between the right dorsal insula and the right middle temporal gyrus was associated with longer years of smoking. The altered insular subdivision dFC can classify smokers from non-smokers with an accuracy of 89.66%, a sensitivity of 96.30% and a specify of 83.87%.

**Conclusions:**

Our findings highlighted the abnormal patterns of fluctuating connectivity of insular subdivision circuits in smokers and suggested that these abnormalities may play a significant role in the mechanisms underlying nicotine addiction and could potentially serve as a neural biomarker for addiction treatment.

## Introduction

Tobacco use stands as the foremost risk factor for cancer-related deaths and years of life lost due to disability (1). Approximately 6 million individuals succumb to smoking-related causes annually, and this figure is projected to escalate to 8 million by 2030 in the absence of effective interventions (1). Although achieving cessation for tobacco use disorder often necessitates multiple attempts, the addictive nature of tobacco use often leads to unsatisfactory treatment outcomes (2, 3). Damage to the insular impedes the compulsion for tobacco consumption, indicating that the insula holds significant potential as a neuromarker for smoking cessation (4–6). Investigating the role of the insula in nicotine addiction offers promise in identifying a target for modifying smoking behavior.

The insula is a highly heterogeneous region, intricately involved in numerous functions through its functional coupling with distinct cerebral areas (7). It can be subdivided into three distinct regions: the dorsal anterior insula (dAI), the ventral anterior insula (vAI), and the posterior insula (pI) (8). The dAI contributes to cognitive control and attention by connecting with the anterior cingulate cortex and dorsolateral prefrontal cortex (9, 10), while the vAI regulates social-emotional processing and autonomic function through its connections with the amygdala and orbitofrontal cortex (10, 11). The posterior insula is linked to primary and secondary somatosensory areas, playing a crucial role in sensory processing (12). Neuroimaging studies utilizing the resting-state functional connectivity (FC) method have revealed abnormal insular networks in smokers. For instance, Compared to non-smokers, chronic smokers exhibit lower FC between the anterior insula(AI) and anterior cingulate cortex(ACC), as well as the ventromedial prefrontal cortex(vmPFC) (13). Longitudinal resting-state FC studies have shown a significant grater in FC between the left anterior insula and left precuneus after treatment in quitters, compared to before treatment (14). Analysis of FC before and after acute withdrawal indicates a significant positive correlation between the right vAI and the dorsal anterior cingulate cortex (dACC) prior to resuming smoking after acute withdrawal. However, no significant correlation was observed after smoking. This suggests that the right vAI -dACC circuit may play a role in maintaining smoking behavior (15). While subdividing insular regions has advanced our understanding of the role of insula in nicotine addiction, most previous studies have relied on the assumption of temporally stationary brain connections and networks during rest (16, 17). Furthermore, existing research has unveiled that smokers and non-smokers exhibit distinctive resting-state indices related to dynamic changes in localized neural activity, such as dynamic regional homogeneity (dReHo) and dynamic amplitude of low-frequency fluctuations (dALFF) (18). Previous studies employing dynamic functional connectivity (dFC) methods have found that smokers experience reduced temporal flexibility and spatiotemporal diversity in brain networks during acute withdrawal (19). However, there is a lack of systematic research on dFC of the insula subregions. Consequently, dynamic regional indexes hold promise as a pioneering neuroimaging biomarker for discerning smoking behavior.

These findings indicate that functional synchronization between spatially distinct brain regions evolves dynamically over the course of resting-state fMRI scans, carrying significant physiological implications for high-level cognitive functioning (20, 21). dFC can capture novel information regarding temporal fluctuations in coupling strength (21, 22). Both spatial and temporal characteristics of the insula were considered, studies have suggested that dFC may offer additional disease-related insights (23, 24). Research has revealed the dFC of the insula, showing its temporal flexibility in terms of function, from the standpoint of dFC of insular subregions, growing evidence was found on brain dysfunction in autism and schizophrenia (25, 26). For instance, one study demonstrated specific abnormal insular connections in autism spectrum disorder, and a linear regression model based on these aberrant dFC patterns was able to predict symptom severity (27). Another study observed alterations in dFC of insular subregions in patients with schizophrenia, and these abnormal dFC patterns normalized after an 8-week antipsychotic treatment (28). However, there are currently no reported studies linking it to nicotine addiction, it remains unclear whether there are specific dFC patterns within insular subregions in smokers with nicotine dependence. Further research is needed to understand the mechanisms and impact of dFC in nicotine addiction. We therefore undertook a comprehensive analysis of correlations with dynamic connectivity patterns of these insular subregions.

dFC can be utilized to observe how nicotine alters dynamic patterns within the brain, thereby potentially identifying biomarkers of addiction or predictors of treatment outcomes. Support Vector Machine (SVM) is a supervised machine learning4/algorithm capable of tackling classification and regression challenges. Consequently, SVM can be integrated with dFC analysis. If proven to be a reliable classifier, it could be employed to predict the onset of nicotine addiction, monitor the progression of the condition, or assess the efficacy of various treatment strategies. The current study aimed to delineate dFC patterns within specific subregions of the insula in individuals with nicotine dependence via a sliding windows method. The SVM was employed to assess whether these abnormal dFC within each insular subregion can accurately distinguish chronic smokers from non-smokers on an individual level. We hypothesized that chronic smokers exhibit aberrant dFC patterns within insular subdivisions and that these atypical fluctuating connections within each insular subdivision may serve as a discriminative neuromaker for classifying smokers with nicotine dependence from non-smokers.

## Materials and methods

### Participants

The current study included 58 male subjects (31 smokers and 27 non-smokers matched for age and education level). Inclusion criteria were: 1) All participants were right-handed, between the ages of 18–45, generally good health. 2)Non-smokers are defined as individuals who have smoked fewer than 10 cigarettes in their lifetime or have never smoked at all. 3)Nicotine-dependent smokers used combustible cigarettes containing nicotine for more than one year and at least four cigarettes per day. Lifetime nicotine dependence diagnosis from smokers was based on the Diagnostic and Statistical Manual of Mental Disorders (DSM-IV) (29) criteria using the Structured Clinical Interview for DSM Disorders (SCID) (30) by two experienced psychiatrists. 4) Measurement of exhaled carbon monoxide (CO) levels was conducted using the Smokerlyzer system (Bedfont Technologies LTD, Rochester, UK) across all participants. It was established that exhaled CO concentrations of ≥6 ppm are indicative of smokers, while a CO content of ≤3 ppm characterizes non-smokers. Smokers and non-smokers were excluded if they: 1) had learning disabilities or central nervous system dysfunctions; 2) had any current or previous major medical or psychiatric disorders; 3) current use intravenous drugs; 4) had undergone current or previous use of electroconvulsive therapy (ECT) or brain stimulation therapies; 5) had a history of head injury with skull fracture or a loss of consciousness for more than 10 minutes; 6) had a family history of psychotic disorder; 7) met substance dependence diagnosis (excluding nicotine dependence for smokers groups); 8) pregnancy or contraindications for MRI. 9) Participants were excluded from the study if head translation exceeded 2 mm or if rotational movement surpassed 2 degrees during MRI scans. Written informed consent was obtained from all participants prior to the study.

### MRI data acquisition

The brain images were obtained using a Siemens Magnetom Trio 3.0 T MRI scanner with an eight-channel head coil at the Magnetic Resonance Center of Hunan Provincial People’s Hospital in China. The scan range encompassed the entire brain, extending from the vertex of the skull down to the base. The acquisition protocol included standard sequences. Gradient echo sequence was used to acquire three-dimensional T1-weighted brain structural images with the following parameters: repetition time = 2,000 ms, echo time = 2.26 ms, field of view = 256 × 256 mm, flip angle = 8°, matrix size = 256 × 256, number of slices = 176, slice thickness = 1 mm. The functional images were acquired using an echo-planar imaging sequence with the following parameters: repetition time = 2,000 ms, echo time = 30 ms, time points = 210, slice thickness = 4.00 mm, flip angle = 90°, matrix = 64 × 64, field of view = 220 × 220 mm^2^. While acquiring fMRI data, especially in fMRI scans, subjects were instructed to keep their eyes closed, not think of anything, and avoid falling asleep.

### MRI data pre-processing

Data processing involved the use of DPABI (31) (http://www.rfmri.org/), SPM (http://www.fil.ion.ucl.ac.uk/spm/), and custom MATLAB code for analysis. The functional images underwent standard preprocessing steps. Initially, the first ten volumes were excluded to account for magnetization equilibration effects and participant adaptation. Subsequently, time delay between slices was corrected and the images were realigned to the first volume for head- movement correction. This process estimated translations and rotations for each volume, indicating head motions. The maximum displacements for all participants were below 2 mm in each axis, and the angular motion was also below 2 for each axis. To control for confounding factors, linear regression was applied, considering six motion parameters along with white matter and cerebrospinal fluid signals. The images were then normalized to the Montreal Neurological Institute standard stereotactic space with a voxel size of 3 mm × 3 mm × 3 mm. Following normalization, a 6-mm full-width at half-maximum Gaussian kernel was used to smooth the images. Finally, temporal bandpass filtering was applied to retain frequencies between 0.01 and 0.08 Hz.

### Dynamic functional connectivity seed-based analysis

**Figure 1:**
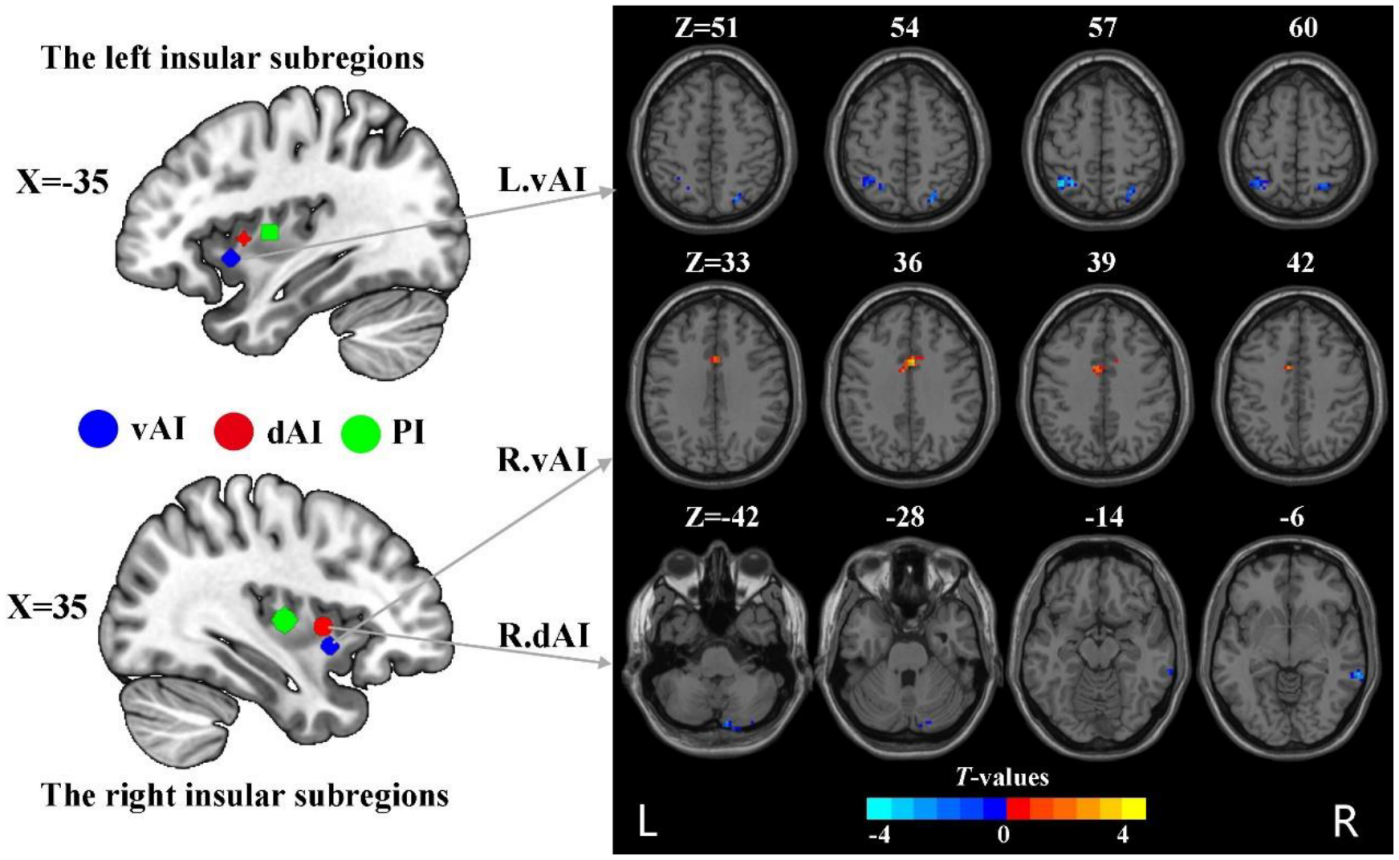
The group difference in dFC variance of insular subregions (voxel *p* < 0.005, cluster *p* < 0.05, Gaussian random field corrected). L, left; R, right; vAI, ventral anterior insula; dAI, dorsal anterior insula.

The subdivisions of insula were defined according to the report by Deen et al (8) (**Figure 1**). Specifically, the insula was divided into three subregions in each hemisphere: the ventral anterior insula (vAI), the dorsal anterior insula (dAI) and the posterior insula (pI). The MNI coordinates of six spherical ROIs with 6-mm radius split from insula were defined as follows: the left vAI (MNI: −33, 13, −7), right vAI (MNI: 32, 10, −6), left dAI insula (MNI: −38, 6, 2), right dAI (MNI: 35, 7 3), left pI (MNI: −38, −6, 5), right pI (MNI: 35, −11, 6).

A sliding-window method with hamming windows was used to assess dynamic functional connectivity (dFC) maps. Following the parameters set in previous studies (32, 33), the window size and the step of slide were set to 50 TRs (100s) and 1 TR (2s), respectively, which resulted in 151 windows. Within each window, the correlation coefficients were transformed to z-values using Fisher’s z transformation. Subsequently, the standard deviation (SD) of dFC over the 151 windows was calculated for each voxel to quantify dFC variability.

### Statistical analyses

The demographic data of the two cohorts, namely the smokers and the healthy controls, were subjected to statistical comparison employing the two-sample t-test and χ2 test. Group differences in dFC variance of insula between smokers and healthy controls were also assessed using the two-sample t-test. To address multiple comparisons, Gaussian random field correction (GRF) was applied with a threshold of voxel p < 0.005 and cluster p < 0.05 (two-tailed). The relationship between significant findings from dFC variance and smoking-related variables (including Smoking Year, Smoking Per Day, and the Fagerström Test for Cigarette Dependence [FTCD]) was evaluated using Pearson correlation. For multiple comparison corrections, the false discovery rate (FDR) was conducted with a threshold p < 0.05.

### Classification analysis and permutation tests

To evaluate whether the variance of functional connectivity (dFC) in insular subregions can distinguish between smokers and non-smokers, a SVM classification model was employed. This machine-learning technique is widely used in classification tasks. The SVM model was trained using a leave-one-out cross-validation (LOOCV) approach on the significant group differences in dFC variance. In this process, the dataset with 58 observations was divided into 58 folds. For each fold, one subject was excluded from the training set to act as a test data point, while the remaining subjects were used to construct and train the classification models. This iterative process ensures that each observation serves as a test data point at least once, allowing for a comprehensive evaluation of the model’s performance. In SVM, a set of features (e.g., functional connections) and corresponding labels (e.g., smoker and non-smoker) are used to train the model. The training process finds the optimal hyperplane that maximally separates the training data. This allows the model to predict the label (group) of new observations based on their derived features. To assess the performance of the classifier, a permutation test was conducted with 10,000 iterations. In each iteration, the class labels were randomly permuted, and the classification accuracy was recalculated. The classification performance was considered reliable if the actual classification accuracy exceeded the 95% confidence interval of the randomly permuted labels. In addition, quantitative measurements, including the area under the receiver operating characteristic curve (AUC), sensitivity, and specificity, were computed. The AUC provides a comprehensive assessment of the classifier’s effectiveness based on the ROC curve. Sensitivity measures the proportion of true positive samples (smokers) correctly identified, while specificity measures the proportion of true negative samples (non-smokers) correctly identified.

## Results

### Sample characteristics

**Table 1** presents the characteristics of 31 male nicotine dependent smokers and 27 male drug-free HC. It includes demographics and nicotine use patterns (smoking duration, daily cigarette consumption, FTCD scale score). There were no significant group differences in age and years of education.

**Table 1:** Participant characteristics of nicotine-dependent smokers and health controls.

	Smokers	HC	T/X	P value
**Sex (M)**	**31**	**27**		
**Age**	**30.32 ± 6.48**	**29.37 ± 5.56**	**0.596**	**0.554**
**Education**	**12.65 ± 2.26**	**13.70 ± 2.60**	**-1.660**	**0.103**
**CO Levels(ppm)**	**15.90 ± 8.98**	**1.52 ± 0.75**	**8.293**	**<0.001**
**Smoking Year**	**8.87 ± 6.63**			
**Smoking Per Day**	**12.26 ± 8.19**			
**FTCD**	**5.74 ± 1.36**			

Values are presented as the mean ± SD. HC, health control.

### The dFC variance difference between smokers and HC

Compared to HC, nicotine-dependent smokers exhibited lower dFC variance between the left vAI and right superior parietal cortex (SPC) and left inferior parietal cortex (IPC) in smokers (*p*<0.05, GRF corrected, **Figure 1** and **Table 2**). We also found smokers showed significantly greater dFC variance between the right vAI and right middle cingulum cortex (MCC). Lower dFC variance between the right dAI and right middle temporal gyrus (MTG) and the right cerebellum Crus2 was observed when compared to smokers and HC. All displayed results were based on the brain region definitions provided by the AAL template.

**Table 2:** The group difference between smokers and HC in dFC variance of insular subdivisions.

Anatomical region	Cluster size (voxels)	Peak coordinates			Peak T-value
		X	Y	Z	
The left ventral anterior insula					
The right Superior parietal cortex	43	24	-69	51	-3.73
The left inferior parietal cortex	48	-39	-57	57	-4.30
The right ventral anterior insula					
The right middle cingulum cortex	55	3	9	36	3.96
The right dorsal insula					
The right middle temporal gyrus	62	66	-39	-6	-4.34
The right cerebellum Crus2	46	9	-84	-42	-3.71

### Brain-behavior analysis

**Figure 2:**
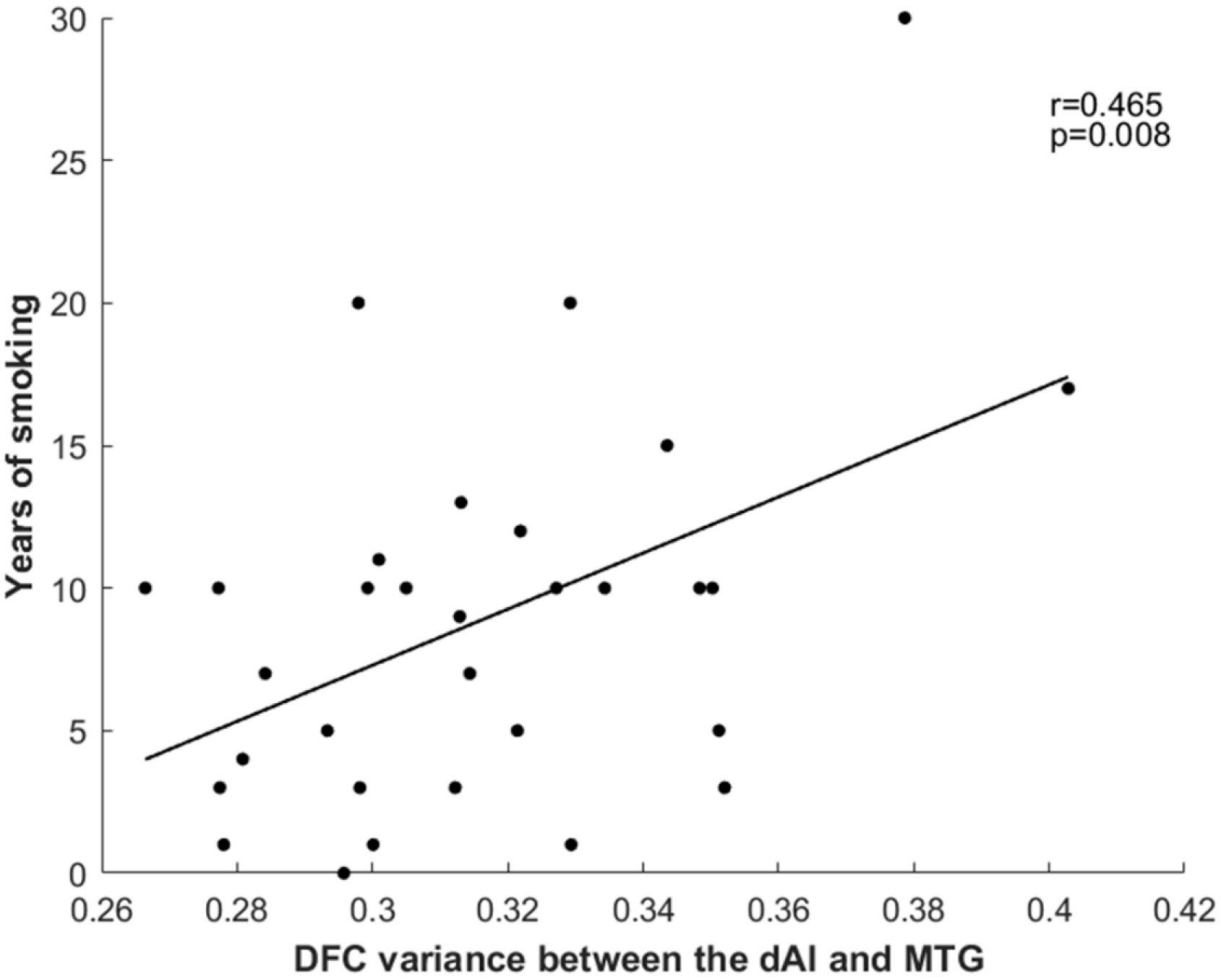
Relationship between years of smoking and dFC variance between the right dAI and right MTG. dFC, dynamic functional connectivity; dAI, dorsal anterior insula; MTG, middle temporal gyrus.

The results revealed a positive correlation between years of smoking and dFC variance between the right dAI and the right MTG (*r* = 0.465, *p* = 0.008) (**Figure 2**). However, no significant correlations were observed with Smoking Per Day or FTCD score.

### Machine Learning Analysis

**Figure 3:**
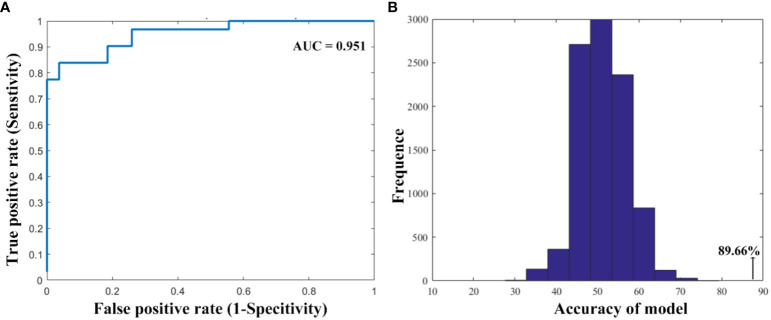
The performance of the classification model. **(A)** The area under the curve and **(B)** the permutation test results.

As shown in **Figure 3**, the SVM classification model can differentiate smokers from HC with an accuracy of 89.66% (AUC = 0.951, sensitivity = 96.30%, specify). The permutation tests revealed a significantly higher classification accuracy based on actual labels in relative to random labels (p < 0.001).

## Discussion

In this study, we investigated abnormal dFC patterns of specific insula subregions in nicotine dependent smokers in relative to HC. Smokers exhibited lower dFC variance between insular subregions and SPC, IPC, MTG, and cerebellum. They also showed grater dFC variance between insular subregions and MCC. Correlation analysis demonstrated that dFC variance between the right dAI and right MTG was positively related with years of smoking. Classification model based on abnormal dFC variance can identify smokers from HC with an accuracy of 89.6%. These findings may provide novel insights into insula functional activity for smokers with nicotine dependence.

We found that smokers with nicotine dependence showed abnormal dFC variance between the vAI and SPC, IPC and MCC compared to HC. The anterior insula, a key hub of the salience network, is implicated in cognitive and affective functions. Notably, smokers exhibit heightened activation in the anterior insula during cue-induced tasks (34). The SPC and IPC are parts of dorsal attention network which involved in visual-spatial attention (35). Previous studies found that altering the circuits between the anterior insula and SPC in smokers plays a crucial role in nicotine dependence and is coupled with action triggered by smoking cues in the left dAI (34). Abnormal dFC variance between the vAI and parietal regions in smokers may be linked to impaired cognitive processing of tobacco-use cues. MCC is recognized for its pivotal role in response selection and feedback-guided decision making (36). Attenuated activation was observed in the anterior insula and MCC in individuals with stimulant use disorder when performing a Paper-Scissors-Rock task (37), which is similar to our results showing lower connectivity of the dAI with MCC over time.

The lower dFC variance between the dAI and MTG and cerebellum in smokers with nicotine dependence were revealed in relative to HC. The dAI is considered to be implicated in cognitive control process, such as detection of novel salient stimuli. The alteration in dFC between the dAI and MTG is lined with a study, which showed lower connectivity of the anterior insula with the MTG in mild cognitive impairment smokers (38). We speculated that this finding may contribute to the effect of tobacco on cognitive control impairment. Additionally, this abnormal dFC of the dAI and MTG in smokers was related with years of smoking. This aligns with the notion that prolonged drug use leads to a shift from reward-directed behavior to habitual and compulsive behavior in individuals with substance use disorder (7, 39), providing additional evidence for the link between alterations in dFC and cognitive control processes. We also found smokers showed lower dFC variance between the dAI and cerebellum. Upon conducting VBM analysis, it was discovered that individuals with substance use disorder showcased a reduction in gray matter volume in the insula and cerebellum regions (40, 41), consistent with our finding. Furthermore, coactivation of the insula and cerebellum were found in individual with substance use disorder during inhibitory control (42).

A classification model based on the dFC variance of the insula demonstrated promising potential for accurately diagnosing individuals with nicotine dependence. Previous studies had established resting-state fMRI as a valuable tool for objectively classifying psychiatric disorders and identifying disease-related neuromarkers at the individual level (43, 44). Traditionally, clinical diagnosis of substance use disorder was primarily reliant on behavioral symptoms (45, 46). Our findings suggested that incorporating dynamic features of insular could potentially offer a novel and objective neural biomarker for addiction treatment.

The present investigation possesses certain constraints. Firstly, the magnitude of the sample was comparatively modest, which could potentially limit the generalizability and statistical power for detecting subtle effects, especially for machine learning analysis, this makes the results less valid. Furthermore, The findings are based on a relatively lenient statistical threshold, which may have a potential bias introduced by multiple corrections across distinct insular subregions. Further studies should explore differences in the insular dFC between smokers and healthy controls using a larger sample size and a more stringent statistical threshold to mitigate potential biases, which is currently in progress. Secondly, our analysis only included male subjects, as the prevalence of male smokers is typically dozens of times that of female smokers. The impact of sex on group differences in dFC of insular subregions should be further explored. Thirdly, recent research indicated that test-retest reliability is low in resting state functional connectivity analysis, Hower, some studies have highlighted the enhanced reliability of dFC variance compared to other FC measures, such as brain states (47, 48). Fourthly, physiological signals, including cardiac and respiratory signals, could introduce artifacts into the research, however, our experiment did not involve the collection of these signals. In addition, given that smokers may have high comorbidity rates with conditions such as alcohol use and depression, it’s essential to note that the current study was unable to estimate the interaction between alcohol use/depression due to the lack of this information. Further studies should delve into exploring the potential effects of comorbid conditions on the dFC. Finally, our findings are derived from a cross-sectional analysis conducted on a single dataset. It is crucial to assess the stability of changes in dFC within specific insular subregions among individuals grappling with nicotine dependence. Considering the well-established importance of the insula in sustaining nicotine addiction, it becomes imperative to delve deeper into the interplay between the dynamic properties of the insula and addiction treatment outcomes, including the risk of relapse, in future research. This can be achieved through comprehensive investigations involving follow-up data and diverse datasets for a more comprehensive understanding of the subject.

## Conclusions

To sum up, the findings of this present investigation demonstrated dynamic features within the various insular subregions. We observed that smokers with nicotine dependence exhibited variance in dFC between the anterior insula and cortical regions, including the IPC, SPC, MCC, and MTG. These abnormal dFC patterns may serve as a diagnostic tool for identifying nicotine addiction. Our findings suggested that the dynamic features of the insula may play a significant role in the mechanisms underlying nicotine addiction and could potentially serve as a neural biomarker for addiction treatment.

## Data availability statement

The raw data supporting the conclusions of this article will be made available by the authors, without undue reservation.

## Ethics statement

The studies involving humans were approved by The Ethical Committee of The People’s Hospital of Hunan Province (The First Affiliated Hospital of Hunan Normal University). The studies were conducted in accordance with the local legislation and institutional requirements. The participants provided their written informed consent to participate in this study.

## Author contributions

AX: Writing – original draft, Methodology, Investigation, Formal analysis, Data curation, Conceptualization. YS: Writing – original draft, Visualization, Software, Methodology, Investigation, Formal analysis, Data curation, Conceptualization. HC: Writing – original draft, Data curation. LL: Writing – original draft, Data curation. PL: Writing – original draft, Data curation. YHL: Writing – review & editing, Validation, Supervision, Project administration, Conceptualization. YGL: Writing – review & editing, Validation, Supervision, Project administration, Conceptualization.
